# T cell activation in biological diagnosis of amoxicillin delayed hypersensitivity

**DOI:** 10.1186/2045-7022-4-S3-P28

**Published:** 2014-07-18

**Authors:** Luc de Chaisemartin, Suzy Lim, Catherine Neukirch, Marguerite Thetis, Pascale Nicaise-Roland, Sandy Peltier, Sylvie Chollet-Martin

**Affiliations:** 1 APHP, Bichat Claude-Bernard Hospital, Immunology Unit, France; 2 APHP, Bichat Claude-Bernard Hospital, Pneumology-Allergology Unit, France

## Background

Delayed hypersensitivity to betalactam drugs is one of the most frequently suspected drug allergies. However, it is sometimes difficult to diagnose. Drug challenges are the diagnostic gold standard, but they are costly and complex to organize. In vitro testing could thus represent a valid alternative. The existence of amoxicillin-specific T cells have long been demonstrated, however few studies have compared the diagnostic performances of the different methods aiming at detecting them, and there is currently no available test validated for diagnostic purpose. In this context, we started a prospective study to establish the relevance of such *in vitro* tests in delayed hypersensitivity to amoxicillin.

## Methods

Peripheral blood mononucleated cells (PBMC) from 3 patients with suspected amoxicillin delayed hypersensitivity and 3 amoxicillin exposed controls were isolated. Cells were then cultivated for 48h with amoxicillin at 1000, 500, and 100mg/L, or medium alone. T cell activation was measured by CD69 expression by flow cytometry and by interferon gamma (INF) ELISPOT. The results were expressed as a ratio between drug stimulated wells and medium alone wells.

## Results

Amoxicillin-exposed controls did not show any detectable lymphocyte activation as measured by CD69 expression or interferon gamma secretion. On the other hand, all patients had detectable activation by at least one test. More specifically, weak INF spots were detected by ELISPOT that were more numerous in amoxicillin-stimulated wells as compared to medium alone. As these signals appeared to be weak, an amplification strategy is currently being set up. CD69 expression analysis appeared to be less sensitive as only one patient had a strong CD69 upregulation, and another one a moderate upregulation.

## Conclusion

These preliminary data are encouraging as to the feasibility of the study. Inclusion of an adequate number of patients will allow us to establish the interest of these tests in amoxicillin delayed hypersensitivity diagnostic strategy. This study will then enlarge its scope to include other molecules.

